# ${\text{NH}}_{4}^{+}$ Association and Proton Transfer Reactions With a Series of Organic Molecules

**DOI:** 10.3389/fchem.2019.00191

**Published:** 2019-04-03

**Authors:** Eva Canaval, Noora Hyttinen, Benjamin Schmidbauer, Lukas Fischer, Armin Hansel

**Affiliations:** ^1^Institute of Ion Physics and Applied Physics, University of Innsbruck, Innsbruck, Austria; ^2^Department of Chemistry and Institute for Atmospheric and Earth System Research (INAR), University of Helsinki, Helsinki, Finland

**Keywords:** NH_4_^+^, chemical ionization, PTR-ToF-MS, association reactions, monoterpenes, acetone, methyl vinyl ketone (MVK), methyl ethyl ketone (MEK)

## Abstract

In this study, we present reactions of ${\text{NH}}_{4}^{+}$ with a series of analytes (A): acetone (C_3_H_6_O), methyl vinyl ketone (C_4_H_6_O), methyl ethyl ketone (C_4_H_8_O), and eight monoterpene isomers (C_10_H_16_) using a Selective Reagent Ionization Time-of-Flight Mass Spectrometer (SRI-ToF-MS). We studied the ion-molecule reactions at collision energies of 55 and 80 meV. The ketones, having a substantially lower proton affinity than NH_3_, produce only cluster ions ${\text{NH}}_{4}^{+}$(A) in detectable amounts at 55 meV. At 80 meV, no cluster ions were detected meaning that these adduct ions are formed by strongly temperature dependent association reactions. Bond energies of cluster ions and proton affinities for most monoterpenes are not known and were estimated by high level quantum chemical calculations. The calculations reveal monoterpene proton affinities, which range from slightly smaller to substantially higher than the proton affinity of NH_3_. Proton affinities and cluster bond energies allow to group the monoterpenes as a function of the enthalpy for the dissociation reaction $NH_{4}^{+}A\to AH^{+}+NH_{3}$. We find that this enthalpy can be used to predict the ${\text{NH}}_{4}^{+}$(A) cluster ion yield. The present study explains product ion formation involving ${\text{NH}}_{4}^{+}$ ion chemistry. This is of importance for chemical ionization mass spectrometry (CIMS) utilizing ${\text{NH}}_{4}^{+}$ as well as ${\text{NH}}_{4}^{+}$(H_2_O) as reagent ions to quantitatively detect atmospherically important organic compounds in real-time.

## Introduction

In the 1990's proton transfer reaction mass spectrometry (Hansel et al., 1995; Lindinger et al., 1998b) using H_3_O^+^ reagent ions became a widely used analytical instrument with applications in environmental science, medical applications, and food technology due to the large amount of volatile organic molecules, which can be quantitatively ionized. H_3_O^+^ undergoes proton transfer reactions with every analyte having a higher proton affinity (PA) than water [PA(H_2_O) = 165.0 kcal/mol (Hunter and Lias, 1998)]. In contrast, ${\text{NH}}_{4}^{+}$ ionization is more specific. Due to the higher proton affinity of ammonia PA(NH_3_) = 204.0 kcal/mol (Hunter and Lias, 1998), exothermic, thus fast, proton transfer reactions between ${\text{NH}}_{4}^{+}$ and analyte are limited to a much smaller number of molecules. If the analyte (A) possesses a proton affinity sufficiently larger than NH_3_, then reaction (1):

(1) $$\begin{array}{l} NH_{4}^{+}+A\to AH^{+}+NH_{3} \end{array}$$

is exothermic and will occur on every collisions, which means that the reaction rate is close to the collisional limit value (Lindinger et al., 1998b). Lindinger et al. (1998a) used proton transfer reactions of ${\text{NH}}_{4}^{+}$ to separate the isomeric molecules α-pinene [PA = 204–209 kcal/mol (Lindinger et al., 1998a; Solouki and Szulejko, 2007)] and 2-ethyl-3,5-dimethylpyrazine [PA > 204 kcal/mol (Lindinger et al., 1998a)], both having a molecular mass of 136 Th, and found that α-pinene was not ionized by ${\text{NH}}_{4}^{+}$. Keough and Destefano (1981) discussed several factors affecting reactivity in ammonia chemical ionization (CI) mass spectrometry. At that time analyte molecules were introduced directly into the ion source where ammonia is present in large excess. The presence of large amounts of NH_3_ and analyte at a typical ion source pressure of one Torr complicates the interpretation of product ion formation due to secondary reactions. Since the introduction of PTR-MS in the early 1990's, a strict separation of the ion source from the reaction region (drift tube) was achieved. This is one key factor why chemical ionization using the PTR-MS design became a quantitative analytical instrument. Keough and Destefano (1981) investigated a series of organic compounds with ${\text{NH}}_{4}^{+}$- chemical ionization. They concluded that analytes having a PA < 188 kcal/mol do not yield useful intensities of ${\text{NH}}_{4}^{+}$(A) adduct ions. Very recently, ammonia chemical ionization was found to be an extremely sensitive method detecting quantitatively first generation oxidized molecules as well as highly oxidized organic molecules with ${\text{NH}}_{4}^{+}$ adduct ion chemistry (Berndt et al., 2018a,b; Hansel et al., 2018). Zhou et al. (2018) applied ${\text{NH}}_{4}^{+}$ chemical ionization in an atmospheric pressure chemical ionization tandem mass spectrometer and investigated the ionization mechanism of molecules with a hydroperoxide moiety.

Here we present detailed results on the mechanism of chemical ionization of eight monoterpenes (C_10_H_16_) by ${\text{NH}}_{4}^{+}$ chemical ionization. Additionally, we investigated the reaction of acetone (C_3_H_6_O), methyl vinyl ketone (MVK, C_4_H_6_O) and methyl ethyl ketone (MEK, C_4_H_8_O) in reactions with ${\text{NH}}_{4}^{+}$. We have chosen the following atmospherically most relevant monoterpenes according to Sindelarova et al. (2014) and Smolander et al. (2014): α-pinene, β-pinene, limonene, ocimene, myrcene, sabinene, 3-carene, and camphene. Ion chemistry was performed at twice the thermal energy (KE_cm_ = 55 meV) and at a somewhat elevated collision energy (KE_cm_ = 80 meV). Additionally, we investigated the effect of absolute humidity on the outcome of the individual reactions. To confirm the experimental results, quantum mechanical calculations on the proton affinities, adduct ion geometries, cluster-bond energies, and reaction enthalpies were performed.

## Materials and Methods

### Experimental Setup and Measurement Procedure

**Figure 1:**
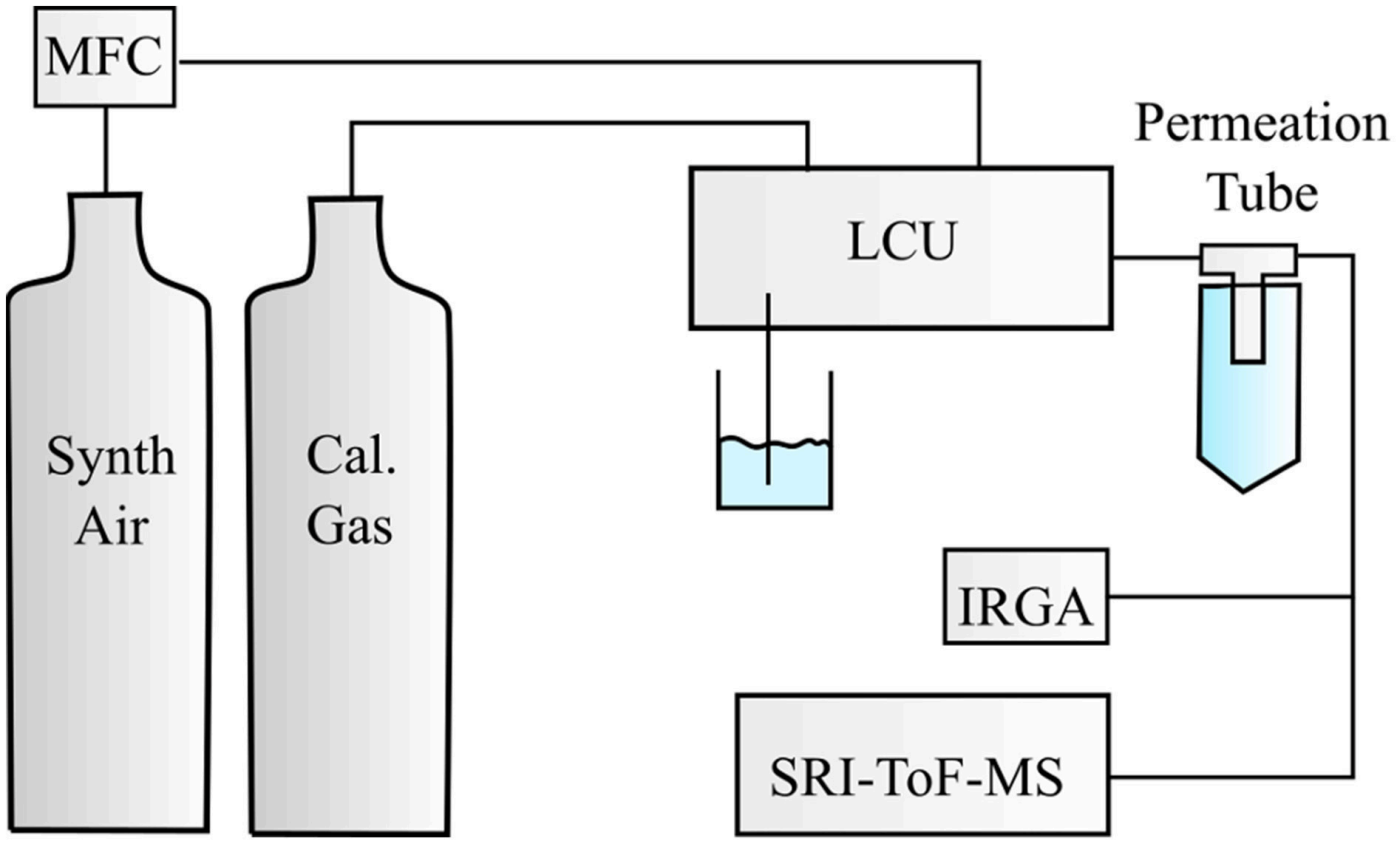
Illustration of the experimental setup. Calibration compounds were either taken from the calibration gas standards or from the diffusion source, which was flushed with a constant synthetic air flow controlled by a mass flow controller (MFC). Different humidity steps were adjusted with the Liquid Calibration Unit (LCU) and monitored by an infrared gas analyzer (IRGA). The humidified synthetic air containing the respective calibration compound(s) was introduced to a selective reagent ion time of flight mass spectrometer (SRI-ToF-MS).

The experimental setup is illustrated in Figure 1. For the investigations of the monoterpenes, a temperature stabilized diffusion source was built. Humidified synthetic air was used to dilute the calibration compound diffusing from the diffusion tube. Humidification of the synthetic air was achieved with a Liquid Calibration Unit (LCU, Ionicon Analytik), which also controlled the carrier gas flow set to 3 slm and the variable liquid water flow. For each calibration compound we measured seven different absolute humidities ranging from 4 ± 3 to 25 ± 3 ppth (parts per thousand).

#### Liquid Calibration Unit (LCU)

The Liquid Calibration Unit (LCU, Ionicon Analytik, Austria) was used to quantitatively evaporate certain amounts of water into the synthetic air stream resulting in absolute humidities in the range of 3–30 ppth. The calibration of the ketones was performed by dynamic dilution of calibration gas standards (Apel Riemer Environmental Inc., Broomfield (CO), USA) in a humidified carrier gas generated by the LCU. Highly water-soluble compounds can be calibrated precisely with the LCU. As monoterpenes are non-polar compounds they are not quite soluble in water. Therefore, we decided to build a temperature-controlled diffusion device, which was combined with the humidified synthetic air stream from the LCU to generate known amounts of monoterpenes in the parts per billion range.

### Temperature Stabilized Diffusion Tube

Each diffusion tube containing several milliliters of the respective liquid calibration compound consisted of a 1/8 inch PFA tubing plug (Parker-Hannifin Corporation, Tucson, USA) connected to a PEEK capillary (Vici Valco, Switzerland) of defined length and inner diameter. The capillary was connected through a gas tight 1/8–1/4 inch tee reducer with the carrier gas stream from the LCU. As described in Fuller et al. (1966), the diffusion rate of a diffusion source is strongly temperature dependent. For this purpose, the diffusion tube was placed in a water bath filled with 50 ml water and wrapped with a heating wire and isolation foam. The water temperature was kept constant at 303 ± 3 K and controlled by a temperature controller (Cal3300, CAL Controls Ltd., Libertyville, USA) connected to a type K thermocouple. The heat capacity of the thermally isolated water bath helped to achieve constant volume mixing ratios (VMR) of individual calibration compounds in the air stream. VMR calculations of the individual analyte in the carrier gas have been performed according to McKelvey and Hoelscher (1957). Assuming that saturation of the head space in the diffusion source has occurred, the diffusion rate r [g s^−1^] of the analyte can be calculated according to Equation (2):

(2) $$\begin{array}{l} r=\frac{D\cdot P\cdot M\cdot A}{R\cdot T\cdot L}\cdot \ln\;\left(\frac{P}{P-P_{s}}\right), \end{array}$$

where D [cm^2^ s^−1^] is the diffusion coefficient, M [g mol^−1^] the molecular weight of the analyte, P [Pa] the total pressure, A [cm^2^] the cross-sectional area of the capillary, L [cm] the length of the capillary, *R* = 8.314·10^6^ cm^3^ Pa mol^−1^ K^−1^ the gas constant, and P_s_ [Pa] the saturation vapor pressure of the liquid analyte. Saturation vapor pressures P_s_ were calculated with MPBWIN v1.43 (© 2000 U.S. Environmental Protection Agency) for the temperature of the diffusion source. Gaussian error analysis estimates an accuracy of the diffusion rate r of ±1% and an accuracy of the obtained volume mixing ratios of ±10–15%. An overview of all values and estimated errors is given in Supplementary Table 2.

### Instrumentation

#### SRI-ToF-MS

The Selective Reagent Ion Time-of-Flight Mass Spectrometer (SRI-ToF-MS) used in this study is based on the PTR-ToF-MS described by Graus et al. (2010) and is adapted for the use with different reagent ions. In principle, it inhibits several advantageous features. The ion source is flushed with ~100 sccm helium, producing only He^+^ and metastable He^*^. The chemical ionization gas NH_3_ (Linde AG, Pullach, Germany) is added later to the source where reactions with He^+^ and He^*^ lead to the formation of ${\text{NH}}_{4}^{+}$ reagent ions. Compared to a standard PTR-ToF-MS, the SRI-ToF-MS is equipped with an ion-funnel located between the ion source and the drift tube having a length of 9 cm. To prevent photochemical reactions in the drift tube, the shape of the ion funnel is constructed in such a way that photons created in the glow discharge ion source don't reach the drift tube. Moreover, the direction of the gas flow through the drift tube and through the ion funnel is opposite to the ion drift direction. This pumping architecture prevents He and CI gases, as well as radicals created in the discharge, from entering the drift region. The metal drift rings used in common PTR-MS instruments are replaced by conductive PEEK rings with a thickness of 6 mm and an inner diameter of 12 mm allowing the measurement of compounds, which catalytically react on metal surfaces. The drift rings are separated by Teflon© spacers of 6 mm thickness. In the present study, the SRI-ToF-MS was operated at 2.3 mbar drift pressure and 35°C drift temperature. The drift voltage was varied between 250 V (E = 27.8 V cm^−1^) and 400 V (E = 44.4 V cm^−1^) resulting in an E/N value of 51 and 81 Td, respectively. E is the electric field strength and N the gas number density (1 Td equals 10^−17^ V cm^2^). Data processing was performed with an adapted version of the data processing routine described in Breitenlechner et al. (2017) and further data analysis was done with Matlab2018a©. Subsequently, to compensate variations in the reagent ion signal and the mass-depended ion transmission in TOF mass spectrometers, product ion signals (e.g., compound i being detected at mass m_i_) measured in counts per second (cps) were duty-cycle corrected (dcps; $dcps\left(i\right)=cps\left(i\right)\cdot \sqrt{100/m_{i}}$) and normalized to 10^6^ cps of ${\text{NH}}_{4}^{+}$ (normalized counts per second, ncps). To study collision induced dissociation (CID) of adduct ions, we ramped the extraction voltage applied to the lenses in the ion transfer region located between the drift tube and the mass spectrometer. For an E/N of 51 Td in the drift tube, we ramped the extraction voltage in 5 V steps between 15 and 25 V. For an E/N of 81 Td the extraction voltages were changed between 20 and 30 V. The errors of the averaged ion signals in each voltage and humidity step are the standard deviations.

### Calculation of Reaction Thermodynamics

In the drift tube, the ions travel as a result of the applied electric field strength E with an increased drift velocity v_d_ through the buffer gas (Lindinger et al., 1998a):

(3) $$\begin{array}{l} v_{d}=\mu \cdot E=\mu_{0}\cdot N_{0}\cdot \frac{E}{N} \end{array}$$

We used ion mobility values μ_0_ of ${\text{NH}}_{4}^{+}$ in N_2_ from Abedi et al. (2014) and adapted them to the gas number density N in our instrument. N_0_ is the gas number density at standard temperature and pressure. Thus, the drift time t of the ions traveling through the drift tube of length l can be calculated by:

(4) $$\begin{array}{l} t=\frac{l}{v_{d}}=\frac{l}{\mu_{0}N_{0}}\cdot \frac{N}{E} \end{array}$$

The reduction of reagent ions [${\text{NH}}_{4}^{+}$] in reactions with the analyte in the drift region is small, thus the reaction can be treated as a pseudo-first order reaction. The density of protonated analytes [AH^+^] is then given according to Lindinger et al. (1998a) by:

(5) $$\begin{array}{l} \left[AH^{+}\right]\approx \left[NH_{4}^{+}\right]\left[A\right]\cdot kt \end{array}$$

Where [A] is the density of analyte A, k the reaction rate coefficient and t the drift time. The ratio $\left[AH^{+}\right]/\left[NH_{4}^{+}\right]$ is proportional to the detected ion signal ratio $i\left(AH^{+}\right)/i\left(NH_{4}^{+}\right)$. Commonly the sensitivity (ε) is defined as the detected analyte signal at a volume mixing ratio of 1 ppbv (parts per billion per volume, 1 ppbv = 10^−9^) normalized to a reagent ion signal i(${\text{NH}}_{4}^{+}$) of 10^6^ cps (Lindinger et al., 1998b). Combining Equations (4) and (5) and using that the volume mixing ratio (VMR) of A is related to [A] by *VMR*(*A*)·*N* = [*A*], the theoretically maximum sensitivity ε_*calc*_ can be calculated as:

(6) $$\begin{array}{l} \varepsilon_{calc}=10^{-3}\cdot k\cdot t\cdot N=10^{-3}\cdot k\cdot \frac{l}{\mu_{0}N_{0}}\cdot \frac{N^{2}}{E} \end{array}$$

We compare the calculated sensitivity with the experimentally observed sensitivity ε_*meas*_. By dynamically diluting either the calibration gas standard or the diffused analyte from the diffusion source, we obtain a known volume mixing ratio VMR(A) of the analyte A. To determine the sensitivity of an analyte A, the contributions of all product ions of the reaction with ${\text{NH}}_{4}^{+}$ must be considered (Cappellin et al., 2012). The measured sensitivity (ε_*meas*_) of a substance A is then given by the slope of a linear fit through the scatter plot of the normalized and duty cycle corrected ion signals of all product ions vs. the volume mixing ratio. The efficiency (eff) of a reaction is then given by

(7) $$\begin{array}{l} eff=\frac{\varepsilon_{meas}}{\varepsilon_{calc}}\cdot 100 \end{array}$$

The center-of-mass kinetic energy *KE*_*cm*_ is calculated according to Lindinger et al. (1998a):

(8) $$\begin{array}{l} KE_{cm}=\frac{m_{b}}{m_{b}+M_{ion}}\left(KE_{ion}-\frac{3}{2}k_{b}T\right)+\frac{3}{2}k_{b}T \end{array}$$

With *KE*_*ion*_ being the mean kinetic energy of ion drifting in the buffer gas:

(9) $$\begin{array}{l} KE_{ion}=\frac{3}{2}k_{b}T+\frac{m_{b}v_{d}^{2}}{2}+\frac{M_{ion}v_{d}^{2}}{2}\;, \end{array}$$

where *m*_*b*_ is the mass of the buffer gas, *M*_*ion*_ the mass of the ions and *k*_*b*_ the Boltzmann constant.

Collisional limiting rate coefficients (k_c_) of ion molecule reactions are calculated according to Su and Chesnavich (1982) and Su (1994) using dipole moments and polarizabilities of the respective analyte (see Supplementary Table 1). Table 1 gives an overview of all reactions. It is worth to mention that we don't have to consider the back reaction of reaction (1) even if the proton affinity of A is only a few kcal/mol higher than the one of NH_3_ in analogy to the H_3_O^+^–formaldehyde reaction system (Hansel et al., 1997). In our case the efficiency of the back reaction is negligible, as in the SRI-ToF the CI gas NH_3_ does not enter the drift region.

**Table 1:** Overview of collisional rate coefficients k_c_, collision energies KE_cm_, calculated sensitivities ε_calc_, measured sensitivities ε_meas_ and reaction efficiencies (eff) at dry and humid conditions.

	**51 Td**							**81 Td**						
				**Dry**		**Humid**					**Dry**		**Humid**	
	**k_c_ [10^−9^ cm^3^/s]**	**KE_cm_ [eV]**	**ε_calc_ [ncps/ppbv]**	**ε_meas_ [ncps/ppbv]**	**eff [%]**	**ε_meas_ [ncps/ppbv]**	**eff [%]**	**k_c_ [10^−9^ cm^3^/s]**	**KE_cm_ [eV]**	**ε_calc_ [ncps/ppbv]**	**ε_meas_ [ncps/ppbv]**	**eff [%]**	**ε_meas_ [ncps/ppbv]**	**eff [%]**
Acetone	4.19	0.055	78.0	3.3	4.2	7.8	9.9	4.0	0.078	46.4	n.d.	–	n.d.	–
MVK	4.22	0.056	78.6	4.6	5.9	8.6	11.0	4.0	0.080	47.0	n.d.	–	n.d.	–
MEK	4.05	0.056	75.5	7.1	9.4	11.5	15.2	3.9	0.080	45.3	n.d.	–	n.d.	–
α-Pinene	2.44	0.057	45.5	14.5	31.9	8.6	19.0	2.4	0.084	28.6	10.7	37.5	10.0	34.8
β-Pinene	2.61	0.057	48.7	13.3	27.3	9.1	18.8	2.6	0.084	30.2	8.7	28.8	9.5	31.3
Camphene	2.48	0.057	46.2	15.6	33.6	11.0	23.8	2.5	0.084	29.0	10.0	34.5	9.7	33.6
3-Carene	2.52	0.057	46.9	15.3	32.6	7.8	16.6	2.5	0.084	29.5	10.6	35.9	9.0	30.5
Limonene	2.56	0.057	47.6	9.6	20.2	5.9	12.3	2.5	0.084	29.8	5.1	17.1	5.3	17.9
Myrcene	2.68	0.057	49.8	10.6	21.3	5.8	11.6	2.7	0.084	31.2	8.9	28.5	7.6	24.4
Ocimene	3.70	0.057	68.8	12.6	18.4	4.8	7.0	3.5	0.084	41.4	12.4	29.8	6.0	14.5
Sabinene	2.76	0.057	51.5	16.3	31.7	13.2	25.6	2.6	0.084	30.7	12.5	40.5	13.0	41.2

*n.d. means not detected. The estimated errors of measured sensitivities are <8%*.

### Quantum Chemical Calculations

Proton affinities are not available for most monoterpenes, thus quantum chemical calculations were performed. Known proton affinities for ammonia, acetone, methyl vinyl ketone and methyl ethyl ketone were compared with our calculations. Additionally, the change of standard enthalpies for the ion-molecule reactions, possible protonation sites and probable ${\text{NH}}_{4}^{+}$ adduct ion structures were calculated. The conformers were sampled using the systematic conformer search algorithm and the MMFF94 force field on Spartan'16 (Wavefunction, 2016). All conformers were optimized at the B3LYP/6-31+G^*^ level of theory using the Gaussian 09 program (Frisch et al., 2009). The geometries of all conformers within 2 kcal/mol of the lowest-energy conformer were optimized and the harmonic frequencies were calculated at the ωB97X-D/aug-cc-pVTZ level of theory using the ultrafine integration grid. The B3LYP/6-31+G^*^ geometry optimization was omitted for some of the protonation products to avoid the breaking and forming of C-C bonds in the cation product. We found the lowest enthalpy protonation products of the monoterpenes by placing the proton to each of the double bond carbons of the compounds at a time. Generally, the most stable protonation product is found when the positively charged carbon of the protonated structure is tertiary. For 3-carene, we found an energetically more favorable structure where the proton was not added to a double bond carbon. Final single-point energies were calculated at the CCSD(T)-F12/VDZ-F12 level of theory for the lowest enthalpy conformers using the MOLPRO program version 2015.1 (Werner et al., 2012, 2015).

### Chemicals

(R)-(-)-limonene (analytical standard), ocimene (mixture of isomers, >90%), camphene (>95%), sabinene (75%), (+)-α-pinene (>99%), and myrcene (analytical standard) were obtained from Sigma Aldrich (Vienna, Austria). (+)-3-carene (>98.5%) and (-)-β-pinene (>99.0%) were purchased from Fluka. Pressured synthetic air grade 5.8 was obtained from Messer (Gumpoldskirchen, Austria), the calibration standard gases were fabricated by Apel Riemer Environmental Inc. (Broomfield, United States). Bottled NH_3_ grade 3.8 was purchased from Linde AG (Pullach, Germany).

## Results and Discussion

### Characteristics of the Diffusion Source

The monoterpenes β-pinene and limonene are also present in our calibration gas standards from Apel Riemer Environmental Inc., Broomfield (CO), USA. They certify an accuracy of typically ±10%. We compared the gas calibration results with our home build diffusion source. The agreement between the estimated sensitivities of the diffusion source and the gas standard differed not more than 25% for these two compounds. As many physical and chemical properties of the investigated monoterpenes are not experimentally determined, we had to rely on calculated values for saturation vapor pressures P_s_ and diffusion coefficients D. Thus, the error of the diffusion source seems very reasonable. To understand the principles of the investigated ion-molecule reactions it is of greater importance that the diffusion rate of the analyte remains constant over the entire measurement period. In our experiment we detected volume mixing ratio drifts of the diffusion source in the range of ± 3% only. Overall we estimate a calibration error of less than ± 30% taking into account also dilution errors from calibrated flow controllers.

### Reagent Ion Distribution

**Figure 2:**
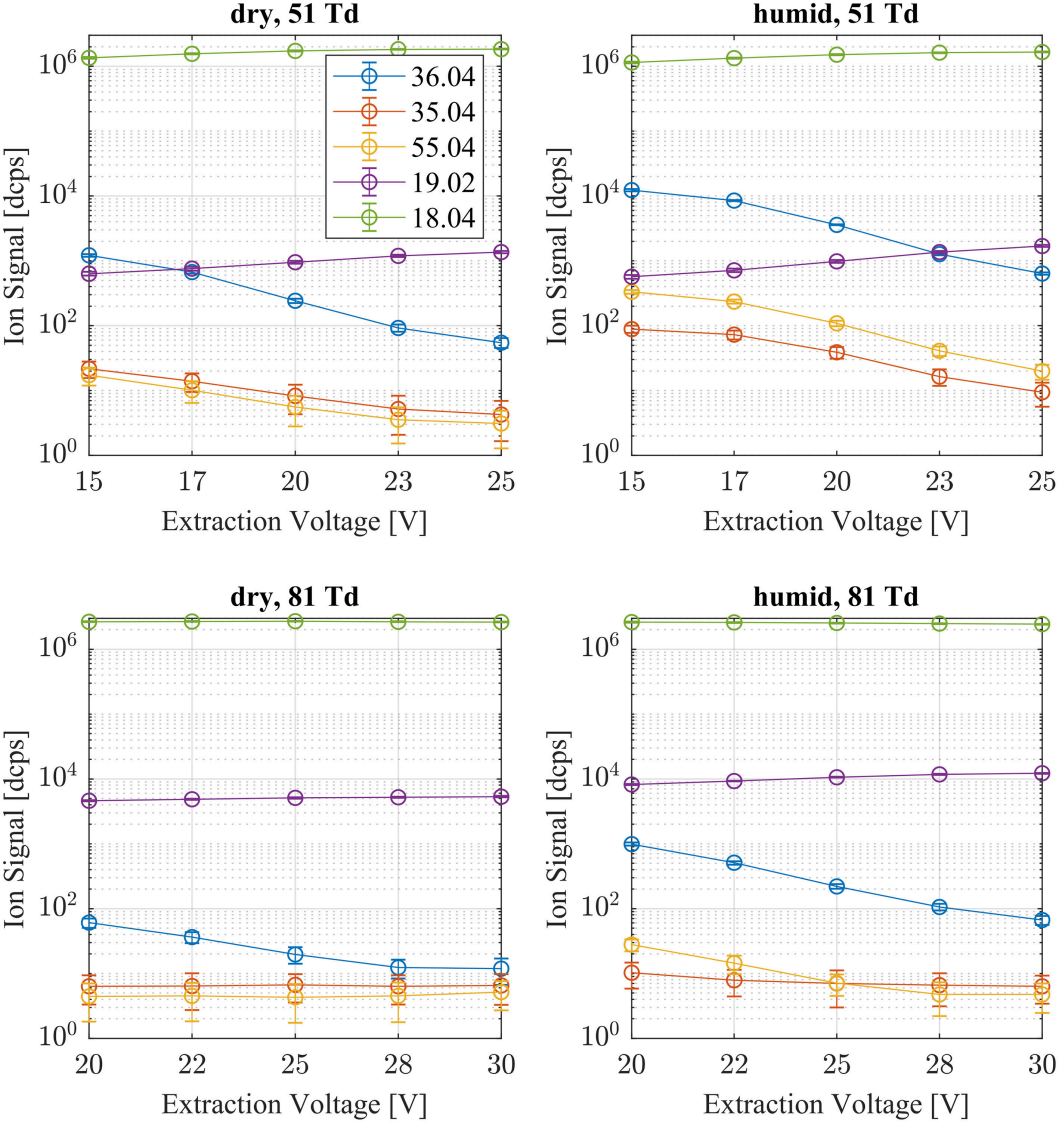
Typical reagent ion distributions are shown at dry (left) and humid (right) conditions as a function of extraction voltage settings at an E/N value of 51 (top) and 81 Td (bottom), respectively. ${\text{NH}}_{4}^{+}$(H_2_O) (*m/z* 36.04), ${\text{NH}}_{4}^{+}$(NH_3_) (*m/z* 35.04), H_3_O^+^(H_2_O)_2_ (*m/z* 55.04), and H_3_O^+^ (*m/z* 19.02) account for <1% of the ${\text{NH}}_{4}^{+}$ (*m/z* 18.04) signal.

Typical reagent ion distributions are shown in Figure 2 as a function of humidity and CID voltage settings. The reagent ion distribution is dominated by ${\text{NH}}_{4}^{+}$. At all voltage settings and humidity steps the ${\text{NH}}_{4}^{+}$ signal dominates with typically 10^6^ dcps. The next prominent reagent ion is the hydrated ammonium ion ${\text{NH}}_{4}^{+}$(H_2_O) (*m/z* = 36.04 Th), which is typically two orders of magnitude lower in intensity even at humid conditions and low E/N settings. The most abundant other “impurity” ions are ${\text{NH}}_{4}^{+}$(NH_3_) (*m/z* = 35.04 Th), H_3_O^+^ (*m/z* = 19.02 Th), NO^+^ (*m/z* = 29.99 Th) and ${\text{O}}_{2}^{+}$ (*m/z* = 31.99 Th). Negligible amounts of H_3_O^+^(H_2_O)_2_ cluster ions are also present. The Gibb's free energies ΔG to dissociate the respective cluster ions at 310 K drift tube temperature are: ${\text{NH}}_{4}^{+}$(H_2_O) ΔG = 13.0 kcal/mol (Meot-Ner and Speller, 1986), H_3_O^+^H_2_O(H_2_O) ΔG = 13.2 kcal/mol (Kebarle et al., 1967), ${\text{NH}}_{4}^{+}$(NH_3_) ΔG = 17.9 kcal/mol (Payzant et al., 1973). In the drift tube we increased the ion energy above thermal applying E/N values of 51 and 81 Td, respectively. Thus, ΔG dissociation energies of the cluster ions become even smaller for higher E/N settings. This is one reason why cluster ion intensities at 81 Td are substantially lower than at 51 Td. The other reason is that the formation of cluster ions is suppressed as a function of ion collision energy in the drift tube (Hansel et al., 1997; Lindinger et al., 1998a). With increasing extraction voltages, the amount of weakly bound cluster ions decrease due to collision induced dissociation (CID) in the ion transfer region. Overall we can conclude that the prevailing reagent ion reacting with the analytes in the drift tube is ${\text{NH}}_{4}^{+}$.

### Reactions of ${\text{NH}}_{4}^{+}$ With Small Ketones

**Figure 3:**
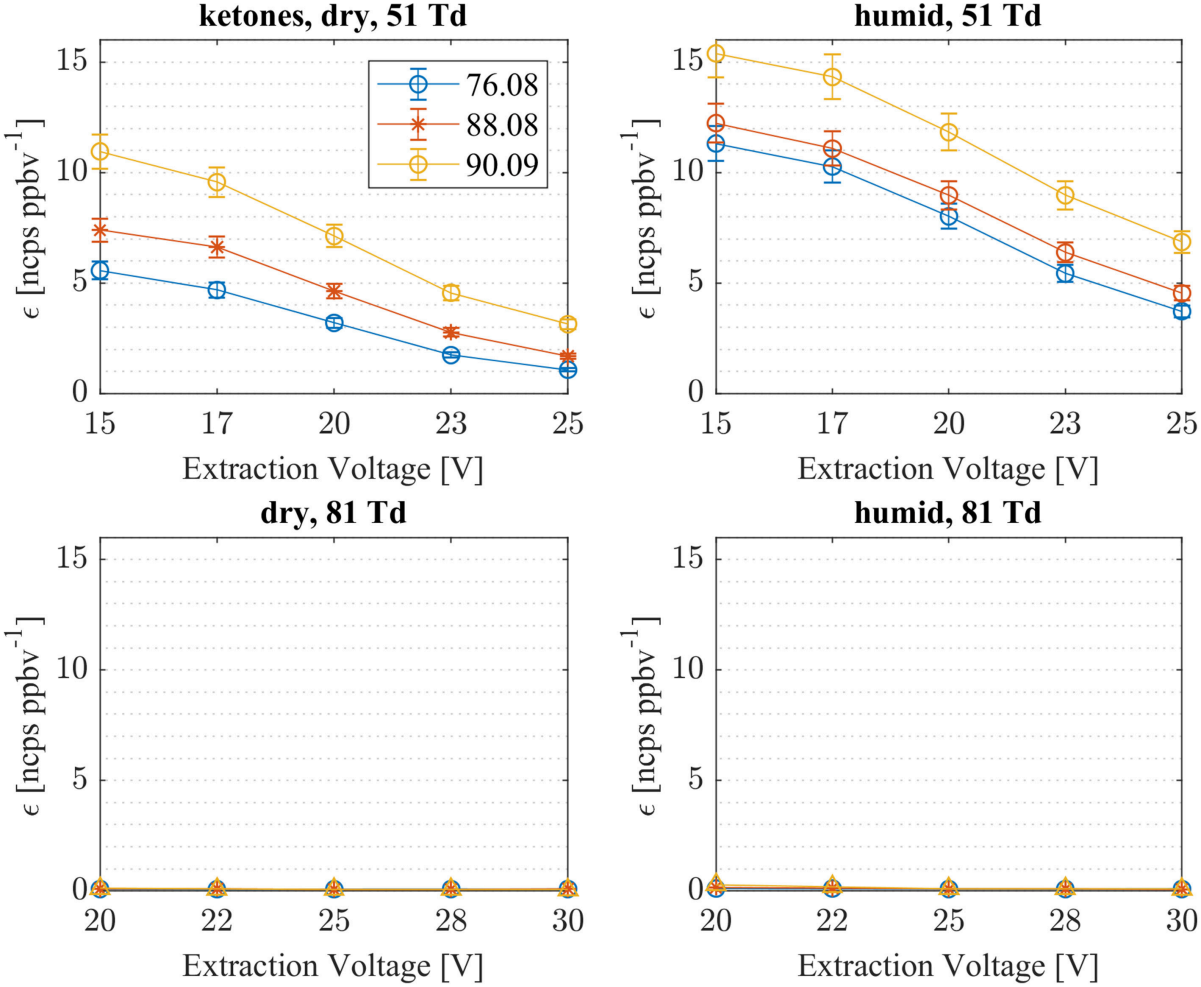
Sensitivities (ε_meas_) of three ketones (acetone: *m/z* 76.08, MKV: *m/z* 88.08, MEK: *m/z* 90.09) for dry (left) and humid (right) conditions as a function of extraction voltages. Cluster ion formation (${\text{NH}}_{4}^{+}$A), which is the only energetically possible reaction, has been observed at low E/N of 51 Td, only.

First, we investigated the reaction of ${\text{NH}}_{4}^{+}$ with acetone, methyl vinyl ketone (MVK) and methyl ethyl ketone (MEK). In Figure 3, measured sensitivities of acetone, MVK and MEK at dry and humid conditions and at two E/N values, 51 and 81 Td, are shown. As illustrated in Figure 3, all ketones are detected as ${\text{NH}}_{4}^{+}$(A) cluster ions: acetone: *m/z* = 76.08 Th, MVK: *m/z* = 88.08 Th, MEK: *m/z* = 90.09 Th. At an elevated E/N value of 81 Td, no cluster ions were observed. This is in agreement with first studies of ammonia chemical ionization of ketones with mass analyzed ion kinetic energy (MIKE) spectrometry (Maquestiaut et al., 1980). No protonated ketones have been observed. For all three ketones, proton transfer reactions with ${\text{NH}}_{4}^{+}$ are energetically unfavorable due to their lower proton affinities compared to ammonia of PA(NH_3_) = 204 kcal/mol [PA(acetone) = 194 kcal/mol, PA(MVK) = 199.5 kcal/mol, PA(MEK) = 197.7 kcal/mol (Hunter and Lias, 1998)]. In SRI-ToF-MS, ${\text{NH}}_{4}^{+}$(A) cluster formation proceeds prevailingly as ternary association reactions under dry conditions. The standard reaction mechanism for ternary (three body or collisionally stabilized) ion-neutral reactions proceeds as follows:

(10) $$\begin{array}{l} A^{+}+B\;{⇋}_{k-}^{k+}\;AB^{{+}^{*}}\overset{M}{\to }\;AB^{+}+M^{{\;}^{*}}, \end{array}$$

where M is a third body, and k^+^ and k^−^ the reactions rates in the respective direction (Ikezoe et al., 1987). Fast ligand switching reactions of type (11), where the water ligand is exchanged by a more strongly bound ketone, could in principle also produce ${\text{NH}}_{4}^{+}$(A) cluster ions but don't contribute much at 2.3 mbar and at elevated collision energies under dry conditions.

(11) $$\begin{array}{l} NH_{4}^{+}\left(H_{2}O\right)+A\to NH_{4}^{+}A+H_{2}O \end{array}$$

Figure 2 shows that ${\text{NH}}_{4}^{+}$(H_2_O) reagent ions are less abundant <1% even at humid conditions and at low E/N values.

**Figure 4:**
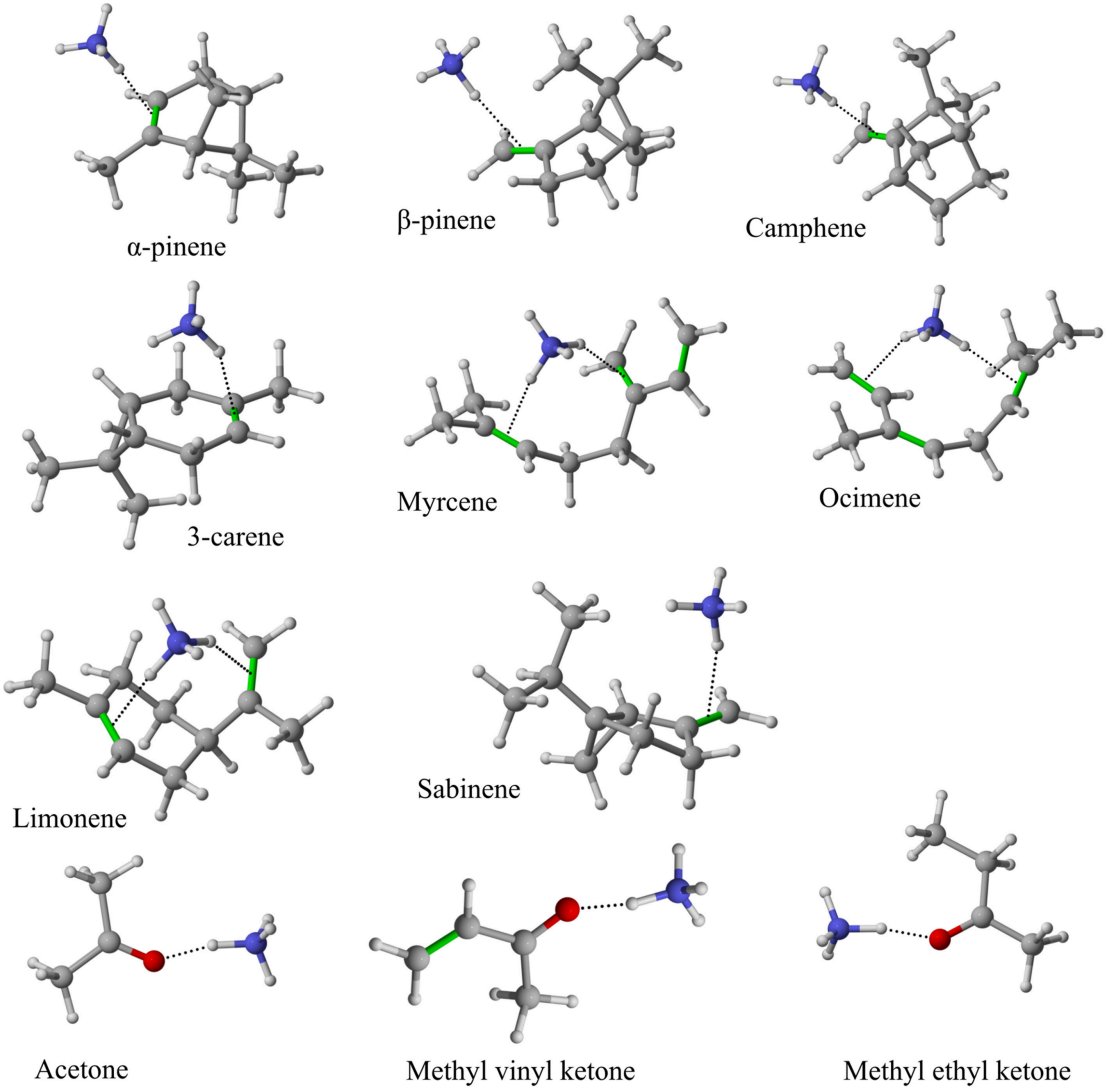
Geometries of the ${\text{NH}}_{4}^{+}$-A cluster ions for all investigated analytes. Color coding: gray = C, white = H, red = O, blue = N. The green bonds indicate C = C double bonds in the analytes.

Table 1 gives an overview of the calculated collisional rate coefficient (k_c_) at the respective collision energy (KE_cm_), the calculated sensitivity (ε_calc_) using k_c_, the measured sensitivity (ε_meas_) and the reaction efficiency (eff) for all compounds studied under dry and humid conditions, and at 51 and 81 Td, respectively. The ketones show reaction efficiencies ranging from 4.2% (acetone), and 5.9% (MVK) to 9.4% (MEK) at dry conditions and a KE_cm_ of 0.055 eV. These efficiencies indicate a rather high effective binary rate coefficient, which is most likely due to the long lifetime of the intermediate (NH_4_A)^+^^*^ against unimolecular decomposition (i.e., k^−^ is small relative to the stabilization rate in collisions with M) for these polyatomic intermediates, which vary with complexity (Johnston, 1966). It is also indicative that the ${\text{NH}}_{4}^{+}$-ketone cluster is strongly bound, as confirmed by quantum chemical calculations, see Table 2 and (Frege et al., 2018). In the lowest energy geometry of the ${\text{NH}}_{4}^{+}$-ketone cluster, one of the hydrogen atoms of ${\text{NH}}_{4}^{+}$ is hydrogen bonded to the oxygen of the carbonyl group (see Figure 4) resulting in typical bond energies of ~ 26 kcal/mol. Adams et al. (2003) studied the association reactions of ${\text{NH}}_{4}^{+}$ with a series of organic molecules using a Selected Ion Flow Tube (SIFT). In this study, Adams et al. (2003) reported that the reaction efficiency for acetone is 22% at 300 K and at 0.5 Torr He pressure. When the efficiency is high as for acetone, this indicates that the ternary reaction is close to pressure saturation, i.e., independent of the He pressure, and in this case only a lower limit to the ternary rate coefficient can be obtained. Earlier studies have shown that the ternary rate coefficient k decreases dramatically as a function of temperature (collision energy) with k α T^−n^, where n ranges from 2 to 3 (Adams and Smith, 1981). An E/N of 51 Td corresponds to 0.055 eV (≈ 600 K). According to Breitenlechner (2011) the fluctuation of the electric field strength along the central axis of the drift tube of the SRI-ToF-MS is within ±10%. Using k α T^−n^ with *n* = 2–3 reduces the reaction efficiency for the ${\text{NH}}_{4}^{+}$-acetone cluster ion formation at 51 Td to 22% × 2^−(2to3)^ = 5.5–2.8% in excellent agreement with our measured reaction efficiency of 4.2%. At an E/N of 81 Td we could not observe any ${\text{NH}}_{4}^{+}$-ketone cluster ions reliably. This is most likely due to the short lifetime of the more excited (NH_4_A)^+^^*^ intermediates at this enhanced collision energy. Under humid conditions the reaction efficiencies of all ketones are increased by about 5% at 51 Td. This increase is somewhat unexpected and might be due to a larger amount of ${\text{NH}}_{4}^{+}$(H_2_O) cluster ions in the drift tube than measured even at the lowest extraction voltage setting of 15 V. Figure 2 shows that about 1% of all reagent ions comprise the hydrated ammonium cluster ions. We would need about 5% of hydrated ammonium cluster ions, which undergo exothermic thus fast ligand switching reactions according reaction (11). Reaction (11) is exothermic for all three ketones. The bond energy of ${\text{NH}}_{4}^{+}$(H_2_O) is 20.6 kcal/mol (Meot-Ner and Speller, 1986) (see Table 1), which is smaller compared to the ${\text{NH}}_{4}^{+}$-ketone bond energies ranging from 25.9 kcal/mol (MEK), 26.4 kcal/mol (acetone) to 27.3 kcal/mol (MVK) (see Table 2). Increasing the extraction voltage from 15 to 25 V decreases the ${\text{NH}}_{4}^{+}$-ketone adduct ions due to CID in the extraction region by 60–70% (Figure 3). In comparison, the ${\text{NH}}_{4}^{+}$(H_2_O) reagent ions show an even more pronounced decrease as a function of extraction voltage (Figure 2). At 25 V, more than 95% of hydrated ammonium cluster ions are lost compared to 15 V extraction voltage. This demonstrates again that cluster ions with a lower bond energy are lost more efficiently in the ion transfer region, supporting our assumption that ${\text{NH}}_{4}^{+}$(H_2_O) cluster ions could be lost even at an extraction voltage of 15 V. In any case our measured amount of ${\text{NH}}_{4}^{+}$(H_2_O) cluster ions (1% compared to ${\text{NH}}_{4}^{+}$) is a lower limit and the enhanced ketone reactivity at humid conditions indicate a higher amount of 5%.

**Table 2:** Overview of the bond energies (BE), reaction enthalpies (ΔH_r_), proton affinities (PA), and protonated structures, calculated at the CCSD(T)-F12/VDZ-F12//ωB97X-D/aug-cc-pVTZ level of theory at 298 K.

**Compound (A)**	**BE [kcal/mol]**	**ΔH_**r**_ [kcal/mol]**	**PA_**calc**_ [kcal/mol]**	**PA_**literature**_ [kcal/mol]**	**Protonated structure**
Ammonia			203.8	204^a^	
Acetone	26.4	35.7	194.5	194^a^	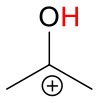
Methyl vinyl ketone (MVK)	27.3	32.9	198.2	199.5^a^	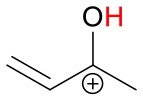
Methyl ethyl ketone (MEK)	25.9	34.0	195.7	197.7^a^	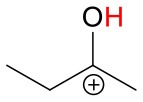
α-Pinene	17.9	15.4	206.3	204–209^b, c^	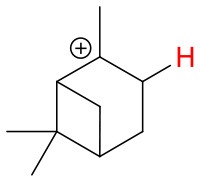
β-Pinene	18.2	13.4	208.7		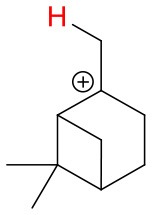
Camphene	18.5	15.2	207.2	205.7^c^	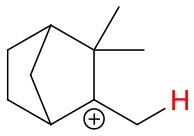
3-Carene	20.6	19.0	205.4		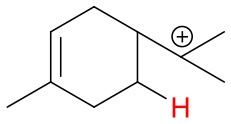
		27.8	196.7		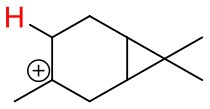
Limonene	22.3	25.0	201.2		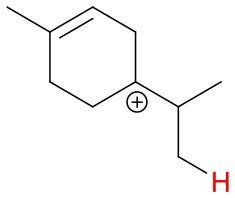
		26.0	200.2		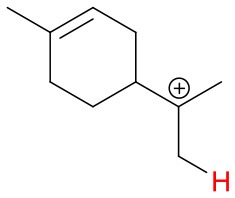
Myrcene	20.9	20.5	204.2		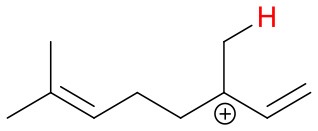
Ocimene	26.0	19.1	210.7		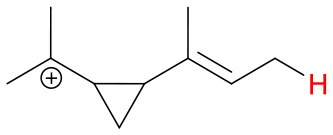
Sabinene	20.6	8.5	215.9		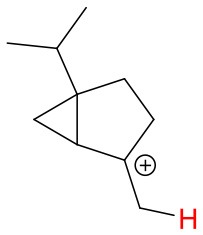

*BE describes the ${\text{NH}}_{\text{4}}^{+}$-A bond energy, ΔH_r_ is the reaction enthalpy of the reaction $NH_{\text{4}}^{+}A\to AH^{+}+NH_{\text{3}}$. We expect errors in computed proton affinities and binding enthalpies to be smaller than 1 kcal/mol. PA literature values are also given if available*.

a *(Hunter and Lias, 1998)*.

b *(Lindinger et al., 1998b)*.

c *(Solouki and Szulejko, 2007)*.

### Reactions of ${\text{NH}}_{4}^{+}$ With Monoterpenes

#### Product Ion Formation

**Figure 5:**
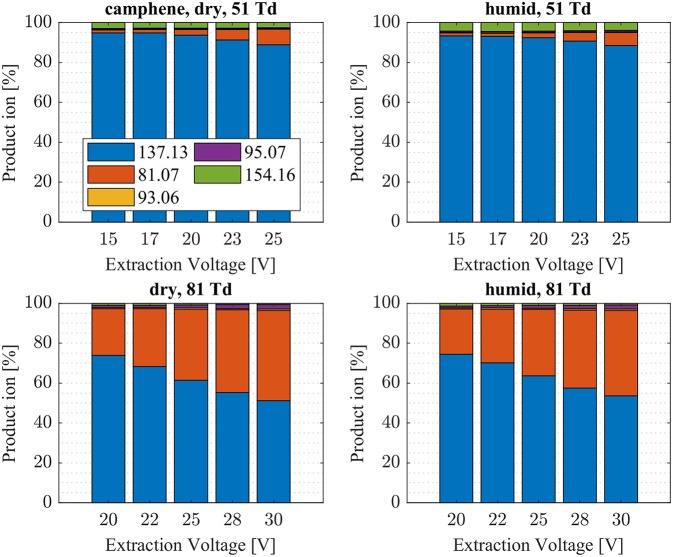
Camphene product ion distributions are shown at dry (3 ± 1 ppth; left) and humid (18 ± 1 ppth; right) conditions as a function of extraction voltage settings at an E/N value of 51 Td (top) and 81 Td (bottom), respectively.

**Figure 6:**
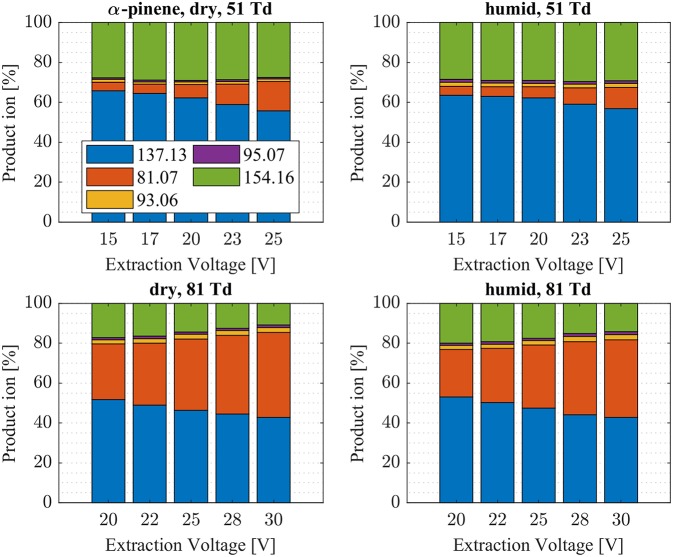
α-pinene product ion distributions are shown at dry (7 ± 1 ppth; left) and humid (26 ± 1 ppth; right) conditions as a function of extraction voltage settings at an E/N value of 51 Td (top) and 81 Td (bottom), respectively.

**Figure 7:**
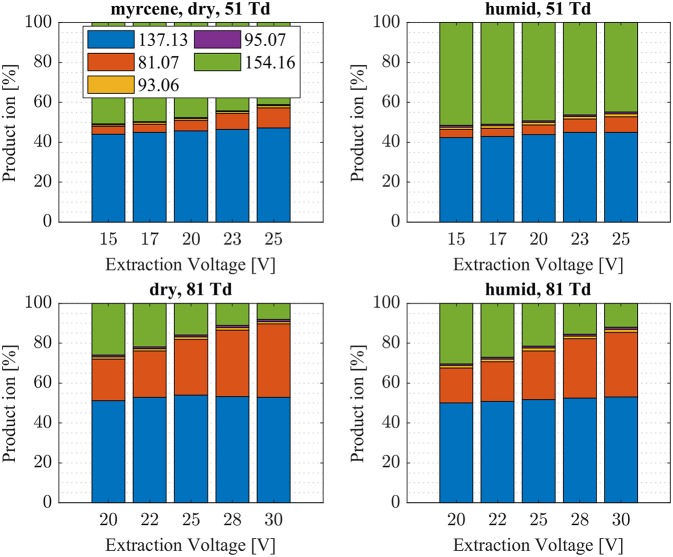
Myrcene product ion distributions are shown at dry (7 ± 1 ppth; left) and humid (26 ± 1 ppth; right) conditions as a function of extraction voltage settings at an E/N value of 51 Td (top) and 81 Td (bottom), respectively.

**Figure 8:**
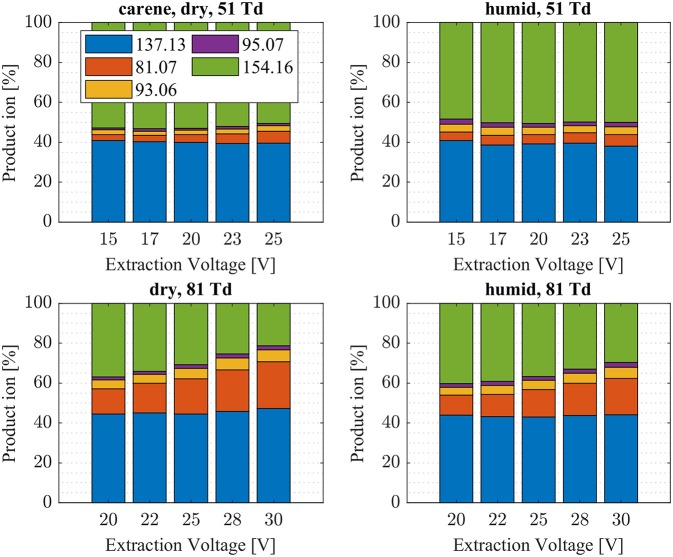
3-Carene product ion distributions are shown at dry (5 ± 1 ppth; left) and humid (26 ± 1 ppth; right) conditions as a function of extraction voltage settings at an E/N value of 51 Td (top) and 81 Td (bottom), respectively.

**Figure 9:**
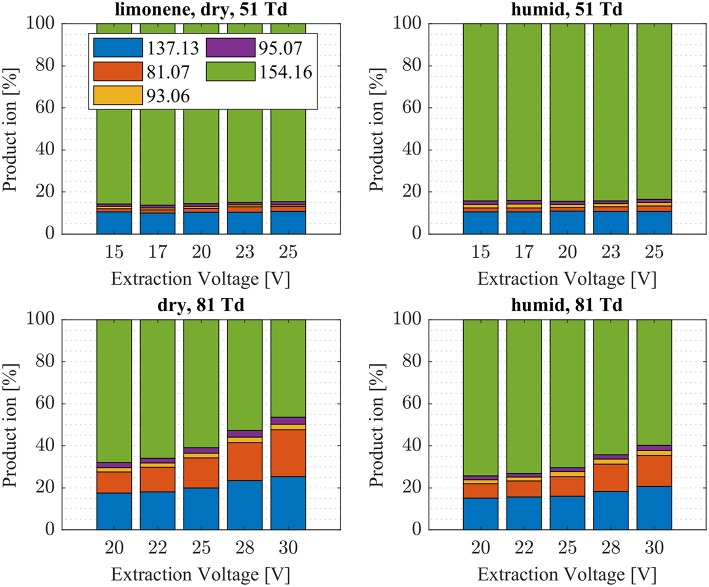
Limonene product ion distributions are shown at dry (7 ± 1 ppth; left) and humid (26 ± 1 ppth; right) conditions as a function of extraction voltage settings at an E/N value of 51 Td (top) and 81 Td (bottom), respectively.

We investigated the reaction of ${\text{NH}}_{4}^{+}$ with eight atmospherically most common monoterpenes at dry and humid conditions, and at two E/N values 51 and 81 Td. In contrast to the measured ketones, for which proton transfer reactions are energetically unfavorable, the proton affinities of the monoterpenes range from 201.2 kcal/mol (limonene) to 215.9 kcal/mol (sabinene), according to our quantum chemical calculations (Table 2). Experimental proton affinities of monoterpenes are rare and exist only for limonene (PA = 209.1 ± 1.2 kcal/mol; Tereza Fernandez et al., 1998), camphene (PA = 205.7 ± 3.2 kcal/mol; Solouki and Szulejko, 2007). For α-pinene (Lindinger et al., 1998a) suggested an upper limit PA < 204 kcal/mol whereas (Solouki and Szulejko, 2007) estimate ~209 kcal/mol. The few experimentally measured proton affinities are in reasonable agreement with our calculations. A list of calculated proton affinities is given in Table 2. Our calculated proton affinity of NH_3_ is 203.8 kcal/mol, which is in excellent agreement with experimental values of 204 kcal/mol (Hunter and Lias, 1998). All investigated monoterpenes, except limonene, have higher proton affinities than ammonia making the proton transfer reaction (1) exothermic. Our results show significant differences in the product ion distribution of the eight studied monoterpenes. As expected we identified the protonated terpene ion C_10_H_16_-H^+^ (*m/z* = 137.13 Th), but also fragment ion C_6_${\text{H}}_{9}^{+}$ (*m/z* = 81.07 Th) and for some monoterpenes additionally small amounts of fragments C_7_${\text{H}}_{11}^{+}$ (*m/z* = 95.08 Th) and C_7_${\text{H}}_{9}^{+}$ (*m/z* = 93.07 Th). Here we record only ion signals, which are detected with relative intensities >1%. Other fragments reported in the literature for H_3_O^+^ chemical ionization using PTR-MS instruments (Wang et al., 2003; Schoon et al., 2004; Tani et al., 2004; Materić et al., 2017) have not been observed. This could be explained by the smaller amount of transferred internal energy using ${\text{NH}}_{4}^{+}$ instead of H_3_O^+^ as reagent ion. The difference in proton affinities between the precursor ion and the respective monoterpene is transferred to the product ion causing fragmentation. Besides proton transfer product ions and corresponding fragment ions, we observe also cluster ions ${\text{NH}}_{4}^{+}$ attached to monoterpenes for all eight monoterpenes. Product ion distributions for the eight monoterpenes are shown in Figures 5**–9** and Supplementary Figures 1–3 at dry and humid conditions, at two E/N values 51 and 81 Td and as a function of extraction voltages.

First, we will discuss the results for dry conditions at an E/N of 51 Td and an extraction voltage of 20 V only. Camphene (Figure 5) and sabinene (Supplementary Figure 1) are the monoterpenes producing only 5% cluster ions ${\text{NH}}_{4}^{+}$-C_10_H_16_ (*m/z* 154.16). The main product ions are protonated monoterpenes C_10_H_16_-H^+^ (*m/z* = 137.13 Th) and the corresponding fragment ion C_6_${\text{H}}_{9}^{+}$ (*m/z* = 81.07 Th). In the case of sabinene (PA = 215.9 kcal/mol) 85% of product ions are protonated sabinene and 10% are found as C_6_${\text{H}}_{9}^{+}$ (*m/z* = 81.07 Th) fragment, while camphene (PA = 207.2 kcal/mol) produces 93% protonated camphene and only 2% C_6_${\text{H}}_{9}^{+}$ fragment ions. Sabinene, having a higher proton affinity compared to camphene, shows a higher amount of fragment ions. Hence more internal energy is generated in the proton transfer channel of sabinene explaining the higher amount of fragmentation. Ocimene (PA = 210.7 kcal/mol) (Supplementary Figure 2) and the two bicyclic monoterpenes β-pinene (PA = 208.7 kcal/mol) (Supplementary Figure 3) and α-pinene (PA = 206.3 kcal/mol) (Figure 6) produce 30–40% cluster ions ${\text{NH}}_{4}^{+}$-C_10_H_16_. The remaining fraction is found prevailingly as protonated monoterpene C_10_H_16_-H^+^ and to a lesser amount at the C_6_${\text{H}}_{9}^{+}$ fragment ion. For the acyclic monoterpene myrcene (PA = 204.2 kcal/mol) (Figure 7) and bicyclic 3-carene (PA = 205.4 kcal/mol) (Figure 8) we detect approximately 50% of the product ion signal at the protonated mass. 3-carene exhibited a slightly dominant cluster ion yield (~54%). Limonene (PA = 201.2 kcal/mol) (Figure 9) shows the highest yield of cluster ions ${\text{NH}}_{4}^{+}$-C_10_H_16_, namely ~85% and the rest is the unfragmented C_10_H_16_-H^+^ ion.

We performed detailed quantum chemical calculations to better understand the ${\text{NH}}_{4}^{+}$ reaction mechanism with monoterpenes. Table 2 gives an overview of calculated proton affinities compared to literature values, the bond energies (BE, in enthalpy) of cluster ions ${\text{NH}}_{4}^{+}$-A, and reaction enthalpies ΔH_r_ of reaction (12).

(12) $$\begin{array}{l} NH_{4}^{+}A\to AH^{+}+NH_{3} \end{array}$$

The structures of protonated compounds are also shown in Table 2. In the cases of 3-carene and limonene we calculated two structures with similar proton affinities, respectively. The lowest enthalpy geometries of all ${\text{NH}}_{4}^{+}$(A) cluster ions are shown in Figure 4. The calculations of the lowest enthalpy conformers of ${\text{NH}}_{4}^{+}$-monoterpene cluster ions reveal that at least one hydrogen of the ${\text{NH}}_{4}^{+}$ forms a hydrogen bond to the C = C double bond with a typical bond energy of ~19 kcal/mol. Some monoterpenes have more than one C = C double bond offering the possibility to form a second hydrogen bond. This is the case for myrcene (BE = 20.9 kcal/mol), limonene (BE = 22.3 kcal/mol), and ocimene (BE = 26 kcal/mol). Bond energies of these monoterpenes are only slightly higher (3–7 kcal/mol) than singly bonding monoterpenes. This is in contrast to calculated ${\text{NH}}_{4}^{+}$ bond energies of compounds containing several carbonyl groups. Introducing a second C = O group increases the stability of the cluster ion considerably (almost 2-fold). Additionally, the position of the second functional group to form an optimal hydrogen bond (with a 180° angle of N-H-O) strongly influences the stability of ${\text{NH}}_{4}^{+}$-carbonyl adduct ions (Frege et al., 2018). To predict the yield of the cluster ion formation channel for the ${\text{NH}}_{4}^{+}$ monoterpene reactions we correlated the fraction of measured cluster ions as a function of monoterpene proton affinities resulting in a correlation coefficient of R^2^ = 0.5 (not shown). The assumption is that monoterpenes with highest proton affinities could perform a quite exothermic direct proton transfer or form an energetically excited (NH_4_-A)^+*^ intermediate that quickly dissociates forming AH^+^ + NH_3_. We therefore expected to find no cluster ion signal for sabinene (PA = 215.9 kcal/mol). As already discussed and shown in Supplementary Figure 1 the cluster ion yield for sabinene is solely 5% under dry conditions.

**Figure 10:**
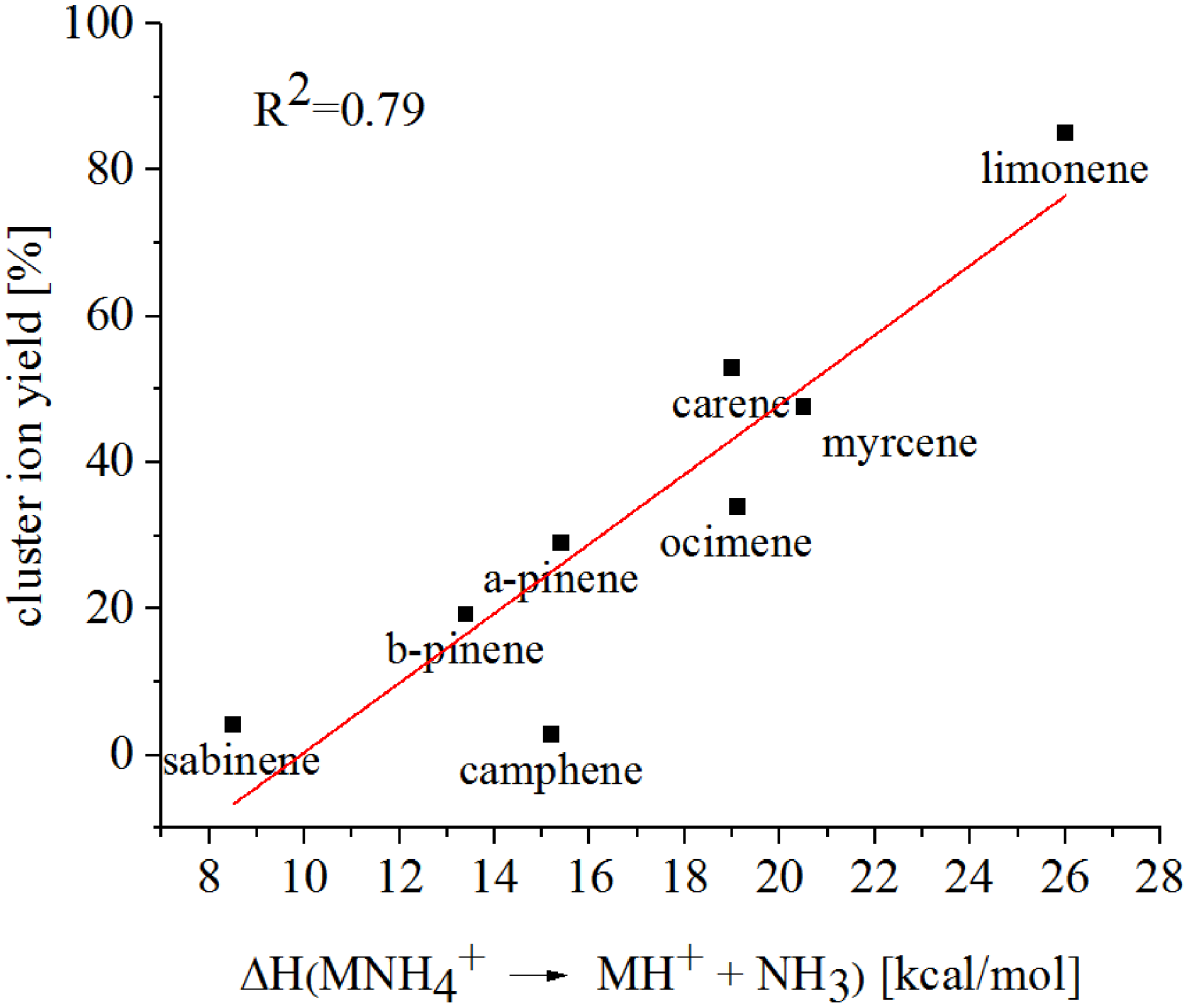
The reaction enthalpy ΔH_r_ of the reaction $NH_{4}^{+}A\to AH^{+}+NH_{3}$, where A is a monoterpene, shows a good correlation with the ${\text{NH}}_{4}^{+}$-A cluster ion yield.

Limonene (PA = 201.2 kcal/mol) has the smallest PA, which is even smaller than NH_3_, and should form exclusively adduct ions only. We observe a cluster ion yield of 85%, which is the highest one of all monoterpenes investigated. But still, there exists a 15% channel at 0.057 eV collision energy producing protonated limonene. At 0.084 eV and dry conditions the yield of the proton transfer channel (including fragment ions) increases to 30% at the lowest extraction voltage (20 V). Increasing the extraction voltage from 20 to 30 V increases this channel even further reaching 50% (Figure 9). This means that the collision energy in the drift tube also leads to additional excitation of the (NH_4_-A)^+*^- intermediate, which is needed for the AH^+^ + NH_3_ channel to become thermodynamically accessible. Increasing the extraction voltage at 0.084 eV collision energy shows a further decrease of the ${\text{NH}}_{4}^{+}$(A) cluster ions, and a gain of AH^+^ and corresponding fragment ions. This is seen not only for sabinene but also for all investigated monoterpenes (Figures 5–9 and Supplementary Figures 1–3). This means that even stabilized ${\text{NH}}_{4}^{+}$(A) cluster ions (and AH^+^ ions) keep more internal energy at higher collision energies in the drift tube and it is then easier to form additional AH^+^ (and corresponding fragment ions) in the ion transfer region through collision induced dissociation (CID). We found the best correlation coefficient (R^2^ = 0.79) to predict the yield of the cluster ion formation channel for monoterpenes (see Figure 10) when the fraction of measured adduct ions was correlated as a function of the reaction enthalpy ΔH_r_ of reaction (12), which is the difference in proton affinities plus the bond energy BE: ΔH_r_ = PA(NH_3_) – PA(A) + BE(${\text{NH}}_{4}^{+}$-A). This is shown in Figure 10, meaning that highest adduct ion yields are formed when compound A is strongly bond to ${\text{NH}}_{4}^{+}$ and the difference in proton affinity is small resulting in smallest internal energy of the (NH_4_A)^+*^ intermediate.

Table 1 gives an overview of the calculated collisional rate coefficient (k_c_) at the respective collision energy (KE_cm_), the calculated sensitivity (ε_calc_) using kc, the measured sensitivity (ε_meas_) and the reaction efficiency (eff) for all compounds studied under dry and humid conditions and at 51 and 81 Td. The monoterpenes show reaction efficiencies ranging from 18.4% (ocimene) to 34.6% (camphene) at dry conditions and a KE_cm_ of 0.057 eV (51 Td). These efficiencies take into account the proton transfer channel, as well as the adduct ion formation channel. Limonene (adduct channel 85%) has a reaction efficiency (eff.) of 20.2%, which indicates a very high effective binary rate coefficient of the cluster channel, which is most likely due to the long lifetime of the intermediate (NH_4_A)^+^^*^ against unimolecular decomposition. Also 3-carene (eff. 32.6%) and myrcene (eff. 21.3%) both having an adduct channel yield of 50% have very high effective binary rate coefficients. Compared to the three ketones (acetone, MVK and MEK), the monoterpenes in general have larger effective binary rate coefficients (cluster ion channel) at 0.057 eV. While in the case of the ketones, no reaction products at all are observed at elevated collision energies (KE_cm_ = 0.084 eV; 81 Td), the adduct ion channel for the monoterpenes is somewhat smaller at this energy compared to 0.057 eV (51 Td) but still quite prominent. The intrinsic difference between the ketone and the monoterpene reaction system is that in the case of the ketones, no rearrangement (exothermic proton transfer) is thermodynamically accessible in the (NH_4_A)^+^^*^ intermediate. The proton in the (NH_4_A)^+^^*^ intermediate, when A is a monoterpene, is not strictly localized at the NH_3_. This can be anticipated comparing the calculated structures of the protonated monoterpenes (Table 2) and the geometries of ${\text{NH}}_{4}^{+}$(A) cluster ions. The delocalization of the charge in the intermediate could enhance the lifetime of the intermediate. Another explanation for the enhanced lifetime of the (NH_4_A)^+^^*^ intermediate is the higher molecular complexity in the case of A being a monoterpene compared to small ketones.

#### Effect of Humidity

The product ion distribution was slightly shifted when changed from dry to humid conditions. More cluster ions (~ 3%) were found under humid conditions (Figures 5–9, Supplementary Figures 1–3). As mentioned before, more water molecules in the sample air lead to an increased formation of ${\text{NH}}_{4}^{+}$(H_2_O) reagent ions. The hydrated ammonium might be able to undergo ligand switching with the monoterpenes, which could explain the slight increase of cluster ions.

#### Selective Detection of Monoterpene Isomers

Since more than a decade different methods have been tested to differentiate the monoterpene isomers using H_3_O^+^-CIMS technology. Müller et al. (2009) coupled a PTR front part to a Triple Quadrupole Tandem MS and a Linear Ion Trap and performed MS/MS studies. The CID spectra of mass selected protonated monoterpene isomers were too similar to specify individual monoterpenes in complex mixtures. Misztal et al. (2012) operated a PTR-MS in an alternating drift voltage mode using 9 ascending and 9 descending voltage steps. The different “time points” of fragmentation of the resulting monoterpene ion signals were used to differentiate between the isomers. Their conclusion was that this method extends the selectivity of the PTR-MS method but cannot compete with the gold standard of GC-MS identification. Here we presented another possibility to separate monoterpene isomers due to their different cluster ion formation in reactions with ${\text{NH}}_{4}^{+}$ reagent ions. We used eight different monoterpenes and found that limonene produces with 85% yield ${\text{NH}}_{4}^{+}$(A) cluster ions while sabinene and camphene have a corresponding cluster ion yield of <5%. In order to predict the yield of the cluster ion formation channel of monoterpenes we propose using the reaction enthalpy of the reaction $NH_{4}^{+}A\to AH^{+}+NH_{3}$ as shown in Figure 10. SRI-TOF-MS allows to switch between different reagent ions such as H_3_O^+^ and ${\text{NH}}_{4}^{+}$ which could be used to extend the selectivity of this CIMS method. But the challenge in separating all monoterpene isomers remains. The fundamental problem arises from the large number of possible isomers that are present at the same time in the real atmosphere.

## Conclusion

In this laboratory study, we investigated the reactions of ${\text{NH}}_{4}^{+}$ with a series of organic analytes (A): acetone (C_3_H_6_O), methyl vinyl ketone (C_4_H_6_O), methyl ethyl ketone (C_4_H_8_O) and eight monoterpene isomers (C_10_H_16_). The reactivity and product ion distribution were studied at two different collision energies and as a function of absolute humidity. Compounds having a lower proton affinity than NH_3_ produced only cluster ions ${\text{NH}}_{4}^{+}$(A). This is the case for the ketones acetone, MVK and MEK, which were observed only at a low collision energy of 0.055 eV. At an elevated collision energy of 0.080 eV no cluster ions of the carbonyls could be detected, meaning that these product ions are formed by association reactions, which are strongly temperature dependent in agreement with earlier observations of Adams et al. (2003). Collision induced dissociation of cluster ions has been studied by varying the extraction voltage applied between the drift tube and the TOF mass analyzer giving a first indication of cluster ion bond energies. Bond energies of cluster ions and proton affinities for most of the compounds used here are not known and have been estimated in the present study by high level quantum chemical calculations. In addition to cluster ion formation, also proton transfer reactions were observed for compounds having a higher proton affinity than that of NH_3_. The monoterpenes have proton affinities ranging from slightly lower to substantially higher than NH_3_. Calculated proton affinities and cluster bond energies allow to group these compounds as a function of the enthalpy for the dissociation reaction $NH_{4}^{+}A\to AH^{+}+NH_{3}$. We find that this enthalpy can be used for the monoterpenes to predict the ${\text{NH}}_{4}^{+}$(A) clusters ion yield. The present study explains product ion formation involving ${\text{NH}}_{4}^{+}$ ion chemistry. This is of importance for chemical ionization mass spectrometry (CIMS) utilizing ${\text{NH}}_{4}^{+}$ as well as ${\text{NH}}_{4}^{+}$(H_2_O) as reagent ions to detect pure hydrocarbon precursor having at least one C = C double bond as well as oxygenated organic compounds in real-time (Berndt et al., 2018a). Here we demonstrated that not only carbonyl compounds, but also hydrocarbons having a higher proton affinity than NH_3_, such as the monoterpenes, can be quantitatively detected with ${\text{NH}}_{4}^{+}$ reagent ions.

## Data Availability

The raw data supporting the conclusions of this manuscript will be made available by the authors, without undue reservation, to any qualified researcher.

## Author Contributions

BS and EC ran the experiments and analyzed the data. NH performed the quantum chemical calculations. EC, NH, BS, LF, and AH took part in the data discussion. LF implemented the raw data analysis software. AH and EC wrote the manuscript. All authors contributed to improvements of the manuscript.

### Conflict of Interest Statement

The authors declare that the research was conducted in the absence of any commercial or financial relationships that could be construed as a potential conflict of interest.
