# Supplementary material for: NH4+ Association and Proton Transfer Reactions With a Series of Organic Molecules
Source: Front Chem. 2019 Apr 3;7:191. doi: 10.3389/fchem.2019.00191 (PMC6457267; doi:10.3389/fchem.2019.00191)
Supplement: Supplementary file 1 [file Data_Sheet_1.docx]

Supplementary Material

# Supplementary Tables

Supplementary Table 1: Polarizabilities (α) and dipole moments (µ_D_) were taken from a) Cappellin et al. (2012), b) predictions ACD/Labs Percepta Platform - PhysChem Module, c) predictions based on Jensen and Kromann (2013), d) CRC Handbook of Chemistry and Physics (2005).

| Compound | α [A^3^] | µ_D_ [Debye] |
| --- | --- | --- |
| acetone | 6.39 a) | 3.11 a) |
| methyl vinyl ketone (MVK) | 8.19 a) | 3.11 a) |
| methyl ethyl ketone (MEK) | 8.17 a) | 2.96 a |
| α-pinene | 17.05 a) | 0.18 a) |
| β-pinene | 18.03 a) | 0.64 a) |
| camphene | 17.3 b) | 0.37 c) |
| 3-carene | 18.11 a) | 0.19 a) |
| limonene | 17.97 a) | 0.49 a) |
| myrcene | 20.02 a) | 0.42 a) |
| ocimene | 18.53 b) | 1.93 c) |
| sabinene | 18.17 a) | 0.91 a) |

Supplementary Table 2: Parameters used to calculate the compound specific diffusion rate.
D is the calculated diffusion coefficient, T is the temperature of the diffusion source, P is the total pressure in the lab, P_s_ is the saturation vapor pressure of the compound, d is the inner diameter of the capillary, L is the length of the capillary and r is the resulting diffusion rate according reaction (2).

| Compound | D [cm^2^/s] | T [K] | P [Pa] | P_s_ [Pa] | d [mm] | L [cm] | r [g/s] |
| --- | --- | --- | --- | --- | --- | --- | --- |
| α-pinene | 0.069±0.007 | 303±3 | 95303±62 | 706±35 | 0.76±0.01 | 4.7±0.05 | 2.6±0.3E-09 |
| β-pinene | 0.069±0.007 | 303±3 | 95303±62 | 450±23 | 0.76±0.01 | 3.7±0.05 | 2.1±0.2E-09 |
| camphene | 0.071±0.007 | 307±3 | 94880±141 | 498±25 | 1.6±0.1 | 3.4±0.05 | 8.5±0.9E-09 |
| 3-carene | 0.069±0.007 | 303±3 | 95050±76 | 380±19 | 0.76±0.01 | 3.7±0.05 | 1.8±0.2E-09 |
| limonene | 0.069±0.007 | 303±3 | 95303±62 | 265±13 | 0.76±0.01 | 4.1±0.05 | 1.1±0.1E-09 |
| myrcene | 0.066±0.007 | 303±3 | 95253±84 | 413±21 | 1.6±0.1 | 3.8±0.05 | 6.3±0.2E-09 |
| ocimene | 0.066±0.007 | 303±3 | 95253±84 | 478±24 | 0.76±0.01 | 3.0±0.05 | 2.6±0.3E-09 |
| sabinene | 0.069±0.007 | 303±3 | 94910±104 | 1280±64 | 1.6±0.1 | 3.1±0.05 | 3.1±0.4E-08 |

# Supplementary Figures

#
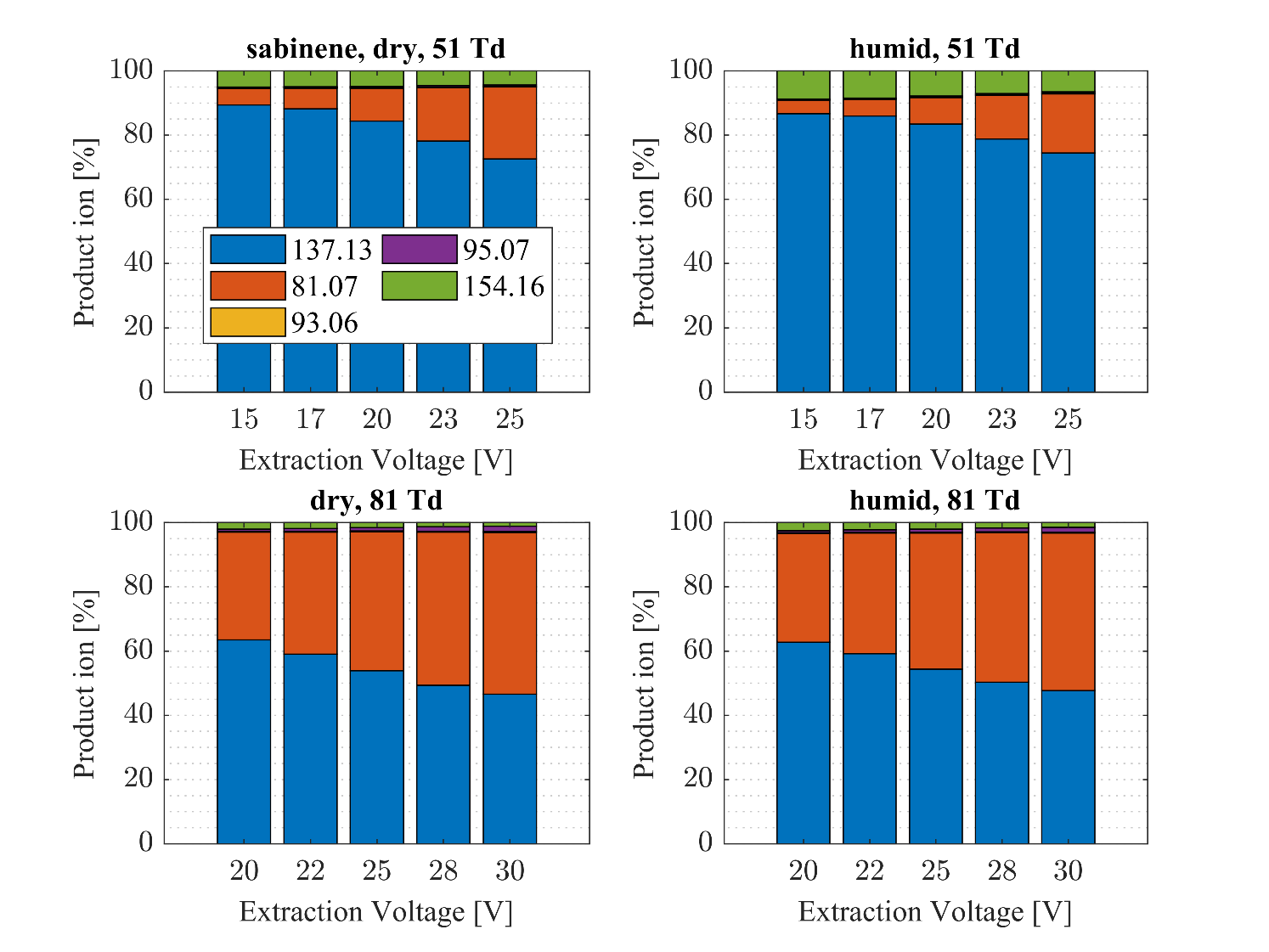


Supplementary Figure 1: Sabinene product ion distributions are shown at dry (3 ± 1 ppth; left) and humid (18 ± 1 ppth; right) conditions as a function of extraction voltage settings at an E/N value of 51 Td (top) and 81 Td (bottom), respectively.


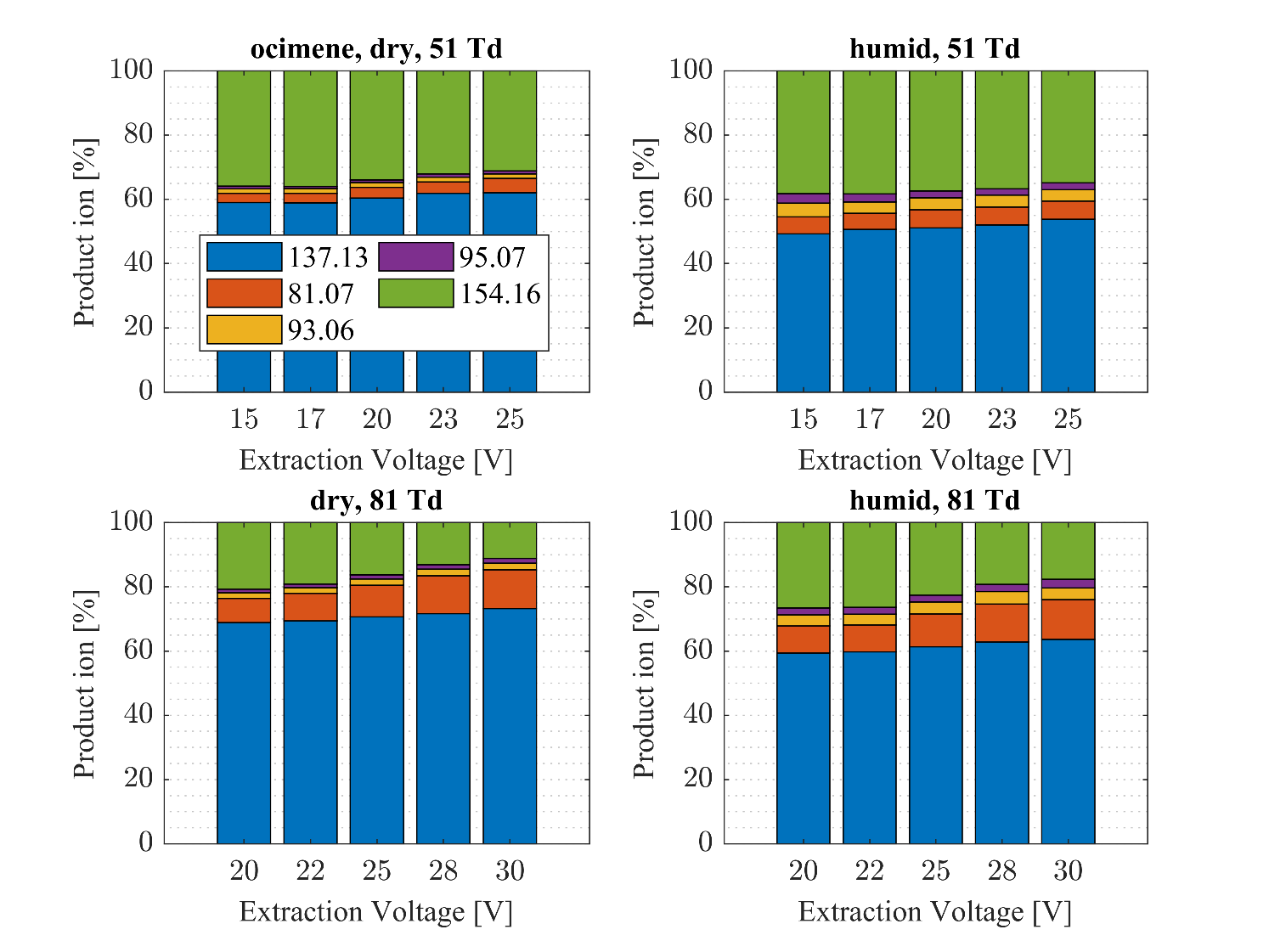


Supplementary Figure 2: Ocimene product ion distributions are shown at dry (6 ± 1 ppth; left) and humid (25 ± 1 ppth; right) conditions as a function of extraction voltage settings at an E/N value of 51 Td (top) and 81 Td (bottom), respectively.


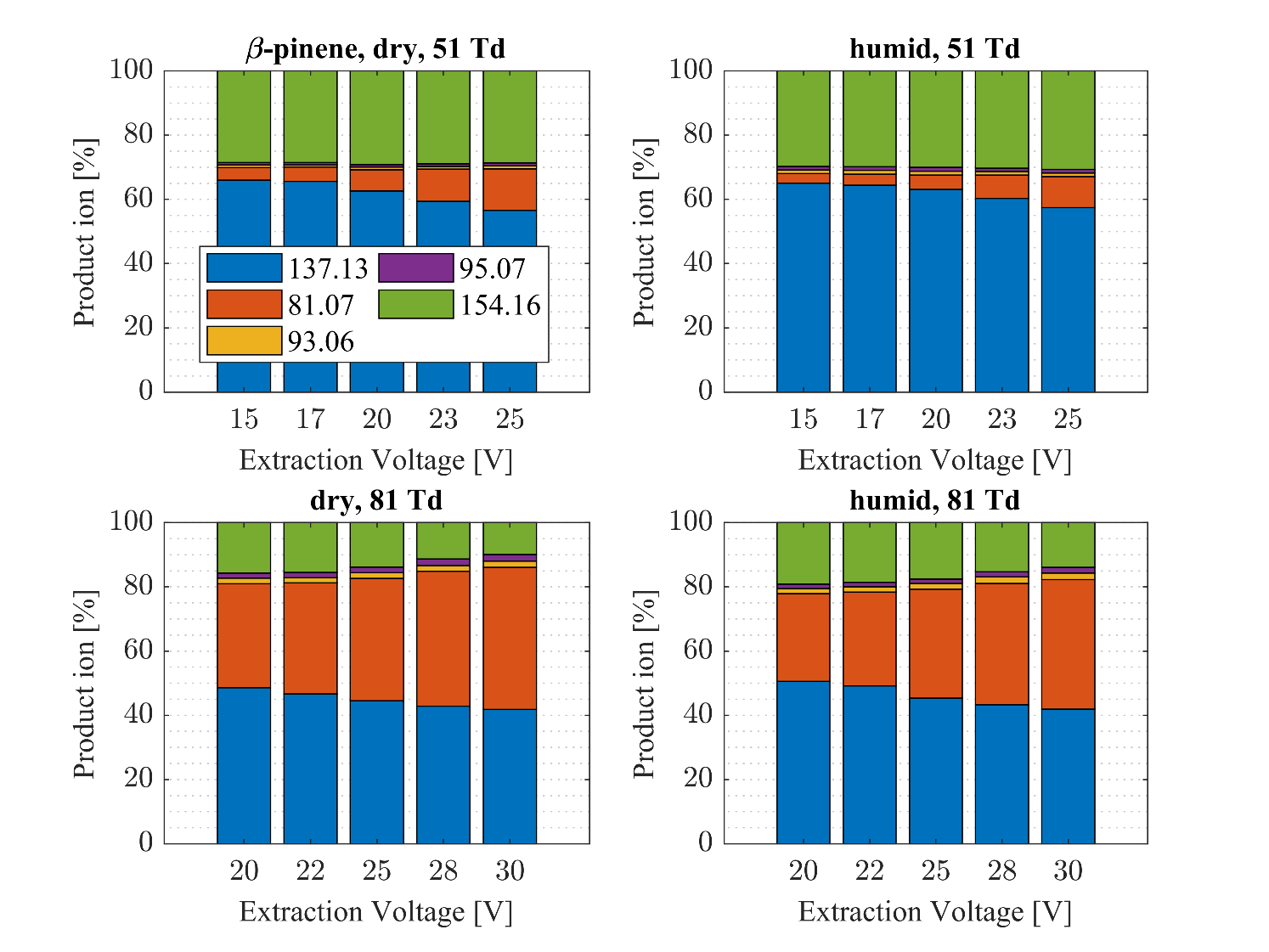


Supplementary Figure 3: β-pinene product ion distributions are shown at dry (6 ± 1 ppth; left) and humid (25 ± 1 ppth; right) conditions as a function of extraction voltage settings at an E/N value of 51 Td (top) and 81 Td (bottom), respectively.

**References**

Cappellin, L., Karl, T., Probst, M., Ismailova, O., Winkler, P. M., Soukoulis, C., et al. (2012). On quantitative determination of volatile organic compound concentrations using proton transfer reaction time-of-flight mass spectrometry. *Environ. Sci. Technol.* 46, 2283–2290. doi:10.1021/es203985t.

David R. Lide, ed., CRC Handbook of Chemistry and Physics, Internet Version 2005, http://www.hbcpnetbase.com, CRC Press, Boca

Jensen, J. H., and Kromann, J. C. (2013). The Molecule Calculator: A Web Application for Fast Quantum Mechanics-Based Estimation of Molecular Properties. *J. Chem. Educ.* 90, 1093–1095. doi:10.1021/ed400164n.
